# Influenza-Associated Intensive-Care Unit Admissions and Deaths — California, September 29, 2013–January 18, 2014

**Published:** 2014-02-21

**Authors:** Patrick Ayscue, Erin Murray, Timothy Uyeki, Jennifer Zipprich, Kathleen Harriman, Catheryn Salibay, Monica Kang, Annie Luu, Rose Glenn-Finer, James Watt, Carol Glaser, Janice Louie

**Affiliations:** 1 EIS officer, CDC; 2 California Department of Public Health, Richmond, CA; 3 Influenza Division, National Center for Immunization and Respiratory Diseases, CDC

The California Department of Public Health (CDPH) conducts surveillance on severe influenza illness among California residents aged <65 years. Severe cases are defined as those resulting in admission to an intensive care unit (ICU) or death; reporting of ICU cases is voluntary, and reporting of fatal cases is mandatory. This report describes the epidemiologic, laboratory, and clinical characteristics of ICU and fatal influenza cases with symptom onset on or after September 29, 2013, and reported by January 18, 2014 of the 2013–14 influenza season. At the time of this report, local health jurisdictions (LHJs) in California had reported 94 deaths and 311 ICU admissions of patients with a positive influenza test result. The 405 reports of severe cases (i.e., fatal and ICU cases combined) were more than in any season since the 2009 pandemic caused by the influenza A (H1N1)pdm09 (pH1N1) virus. The pH1N1 virus is the predominant circulating influenza virus this season. Of 405 ICU and fatal influenza cases, 266 (66%) occurred among patients aged 41–64 years; 39 (10%) severe influenza illnesses occurred among children aged <18 years. Only six (21%) of 28 patients with fatal illness whose vaccination status was known had received 2013–14 seasonal influenza vaccine ≥2 weeks before symptom onset. Of 80 patients who died for whom sufficient information was available, 74 (93%) had underlying medical conditions known to increase the risk for severe influenza, as defined by the Advisory Committee on Immunization Practices (ACIP). Of 47 hospitalized patients with fatal illness and known symptom onset and antiviral therapy dates, only eight (17%) received neuraminidase inhibitors within 48 hours of symptom onset. This report supports previous recommendations that vaccination is important to prevent influenza virus infections that can result in ICU admission or death, particularly in high-risk populations, and that empiric antiviral treatment should be promptly initiated when influenza virus infection is suspected in hospitalized patients, despite negative results from rapid diagnostic tests.

During the 2009 influenza pandemic, LHJs in California were required to report severe cases of influenza to CDPH. Severe case reporting was voluntary after the pandemic until August 2011, when influenza-associated deaths among persons aged <65 years were made reportable. ICU cases among patients aged <65 years remained voluntarily reportable to the state; 57 of 61 LHJs report such cases in real time.* For this report, a fatal case was defined as a death occurring in a California resident aged <65 years who had a positive test for influenza and clinical signs and symptoms compatible with influenza with onset on or after September 29, 2013, and reported by January 18, 2014. An ICU case met the same definition as a fatal case, but occurred in a patient hospitalized in an ICU who had not died by January 18, 2014.

Acceptable laboratory confirmation methods for influenza included testing respiratory specimens by reverse transcription–polymerase chain reaction (RT-PCR), direct-fluorescent antibody staining, viral culture, or rapid influenza diagnostic tests. Cases were reported by providers, hospitals, medical examiners, and coroners to LHJs, which then reported cases to CDPH. CDPH sought and abstracted data from autopsy and medical records for fatal cases and reviewed available data for all severe cases for the 2013–14 season received through January 18, 2014. Data reviewed included patient demographics, clinical course and treatment, underlying medical conditions, influenza vaccination status, and laboratory testing. Comparisons with previous influenza seasons were made by using CDPH influenza data from the period 2009–2013. Population estimates were derived from the California Department of Finance for relative risk (RR) calculations comparing the group aged 41–64 years with younger age groups in aggregate.

## Epidemiologic Characteristics

**FIGURE 1:**
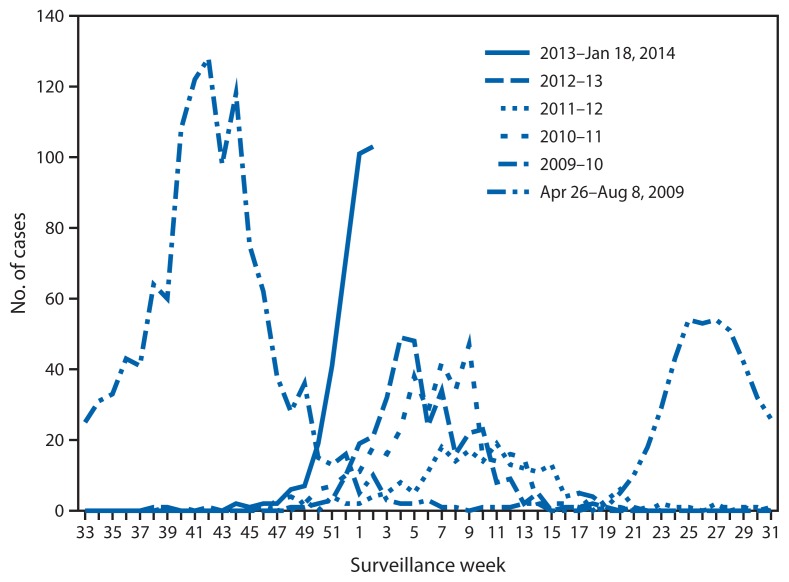
Number of cases of severe influenza,* by week of symptom onset — California, April 26, 2009–January 11, 2014^†^ * Severe cases of influenza are defined as influenza infections resulting in intensive care unit (ICU) admission or death. ^†^ ICU cases from three large local health jurisdictions have not been fully reported yet for the 2013–14 influenza season; for comparability, their ICU data are excluded from all years in this figure. Only cases occurring through January 11, 2014, are included because reporting for the cases with onset in the week ending January 18, 2014 was incomplete at the time of this report.

As of January 18, 2014, 405 ICU and fatal influenza cases had been reported from 41 (67%) of 61 LHJs† in California; symptom onset dates were October 20, 2013–January 15, 2014. The largest number of severe cases (103) by week of symptom onset occurred during the week ending January 11, 2014. These represent the highest cumulative number of severe cases at this point in the influenza season and the highest number of new cases in a single week since the 2009 H1N1 pandemic (Figure 1).

**FIGURE 2:**
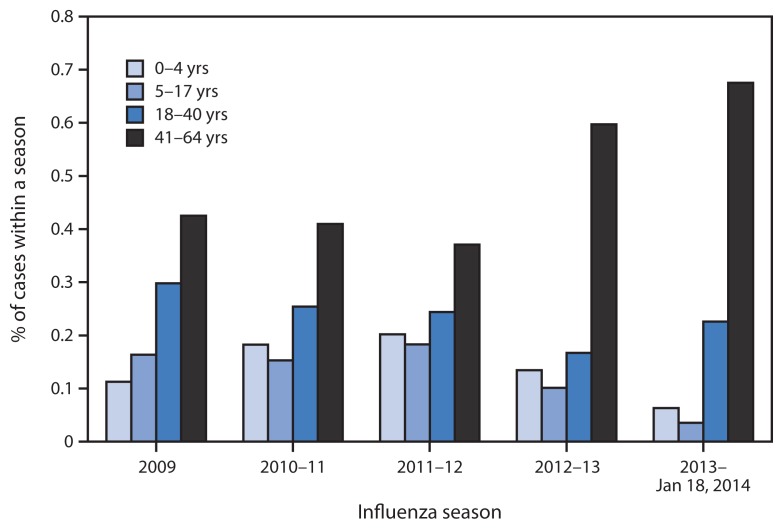
Percentage of severe* influenza cases, by age group, across influenza seasons — California, 2009–January 18, 2014 * Severe cases of influenza are defined as influenza infections resulting in intensive-care unit admission or death.

Three fatal influenza cases and 36 ICU cases were among children aged <18 years, including one fatal case and 24 ICU cases among those aged <5 years (Table). Among the 94 fatal cases and 311 ICU cases, 72 (77%) and 195 (63%) were among persons aged 41–64 years, respectively (Figure 2). These are higher proportions than in any season for which data were compared (2009 pandemic to present; p<0.03). Persons in the 41–64 years age group had six times the risk for death (RR = 6.0; 95% confidence interval [CI] = 3.7–9.6) and almost four times the risk for ICU admission (RR = 3.8; CI = 3.1–4.7) versus those aged ≤40 years. Of 25 pediatric ICU and fatal cases, the proportion (6%) among children aged 0–4 years is the lowest observed per season since the 2009 pandemic (p<0.03).

## Laboratory Characteristics

All 94 fatal cases were associated with influenza A virus; subtyping was performed on respiratory specimens from 77 (82%) patients, and all specimens were identified as pH1N1 virus. Of 311 ICU cases, 303 (97%) tested positive for influenza A virus, and eight (3%) tested positive for influenza B virus. Of ICU cases testing positive for influenza A, 165 (54%) were subtyped, and all were identified as pH1N1 virus.

Results of rapid influenza diagnostic tests§ were reported in medical records of 24 (26%) of the 94 fatal cases. Ten were negative results, indicating a false-negative rate of 42%, compared with RT-PCR.

## Clinical Characteristics

Of the 94 patients who died, 80 (85%) had sufficient medical history reported to determine whether they had preexisting conditions that put them at high risk for influenza complications as defined by ACIP (1). A comorbid condition predisposing to severe influenza was identified in 74 (93%) of these 80 patients with fatal illness. One fatal case occurred in a pregnant woman who had other preexisting medical conditions. The most commonly noted ACIP comorbid conditions were diabetes mellitus (20 cases [25%]), chronic obstructive pulmonary disease (16 [20%]), asthma (11 [14%]), and morbid obesity (body mass index ≥40) (11 [14%]). Of the six patients with no known comorbid condition predisposing them to complications from influenza, as defined by ACIP, three (50%) were obese, with body mass indices of 30–39.

Only six (21%) of 28 decedents whose vaccination status was known had documentation of receipt of 2013–14 seasonal influenza vaccine ≥2 weeks before symptom onset. Ten (11%) of the 94 patients who died were not hospitalized. Hospitalized patients who died were admitted to the ICU a median of 6 days after symptom onset (range = 0–56 days) and spent a median of 5 days in the ICU (range = 0–22 days). Of 65 fatal cases among persons for whom clinical information was available, 60 (92%) patients underwent endotracheal intubation and received mechanical ventilation.

In 80 fatal cases for which antiviral treatment information was available, neuraminidase inhibitors (e.g., oral oseltamivir or inhaled zanamivir) were prescribed for 62 (78%), with 57 (92%) of these patients receiving oral oseltamivir. Of 58 fatal cases with known dates of antiviral therapy, 27 patients (46%) received antiviral treatment on or before their hospital admission date. Of 47 patients with fatal illness and known symptom onset and antiviral therapy dates, eight (17%) received neuraminidase inhibitors within 48 hours of symptom onset.

### Editorial Note

Surveillance for severe influenza can support an assessment of the severity of influenza seasons, identify populations most affected, and identify emerging influenza viruses that can cause substantial morbidity and mortality. Surveillance for severe influenza (i.e., fatal and ICU cases) also enabled CDPH to identify unusual characteristics of influenza activity early in the 2013–14 influenza season. In contrast with previous seasons, a higher proportion of severe cases in the current season in California were reported among adults aged 41–64 years, and a lower proportion among children aged 0–4 years. In addition, severe cases were being reported in higher numbers and earlier in the season than in any season since 2009. The majority of patients with fatal illness tested positive for pH1N1 virus, suffered from comorbid conditions predisposing them to severe influenza complications, and had not received 2013–14 seasonal influenza vaccine.

The proportion of severe cases among children is the lowest observed since the 2009 pandemic, when data collection for all severe cases in persons aged <65 years began, although high rates of hospitalization were observed among this age group in 2009 (2). These data from the 2013–14 influenza season demonstrate that patients aged 41–64 years were at relatively higher risk for influenza than in previous recent influenza seasons. The reason for this difference is unknown and might include virologic factors or a relative lack of population immunity in this age group because of low rates of either vaccination or prior exposure. The difference might also reflect bias resulting from reporting changes across time. National hospitalization rates for laboratory-confirmed influenza for this season also are following an unusual age distribution, with 61% of hospitalizations occurring among persons aged 18–64 years (3).

The majority of fatal cases reviewed occurred among persons who had underlying conditions predisposing them to severe influenza and who had no record of having received 2013–14 influenza vaccine. Clinicians should make influenza vaccination a priority for all patients, and early diagnosis and treatment of influenza-like illness should be a priority in the care of patients with preexisting conditions recognized by ACIP as increasing the risk for influenza complications (4).

This review of severe cases has highlighted potential gaps in clinical care of critically ill patients with suspected influenza. These data support previous findings that rapid influenza diagnostic tests have inadequate sensitivity in identifying influenza virus infection compared with RT-PCR (5,6). Additionally, even when RT-PCR is used, clinicians should consider testing lower respiratory tract samples (e.g., bronchoalveolar lavage or endotracheal aspirate) among undiagnosed critically ill patients because upper respiratory samples can test negative among patients with severe lower respiratory tract disease (7).

Approximately 54% of hospitalized patients with fatal illness did not receive antiviral treatment at hospital presentation. Neuraminidase inhibitors have an excellent safety profile and empiric treatment with a neuraminidase inhibitor should be initiated as soon as possible for any hospitalized patient with suspected influenza (8). For outpatients with high-risk conditions and persons with progressive disease who are not being admitted, antiviral treatment is also recommended (9).¶ Observational studies have also reported modest clinical benefits when antiviral treatment is started late in the course of illness, which indicates that even patients admitted late in the course of illness should receive antiviral treatment (10). Either oral oseltamivir or inhaled zanamivir are recommended for treatment of suspected or confirmed influenza ( http://www.cdc.gov/flu/professionals/antivirals/summary-clinicians.htm ). Inhaled zanamivir should not be used for patients who are severely ill with influenza or intubated. For severely ill patients with influenza who cannot receive oral oseltamivir or inhaled zanamivir, intravenous zanamivir, an investigational drug, can be considered.** Oseltamivir resistance is low among circulating influenza viruses in the United States.

The findings in this report are subject to at least four limitations. First, because the analysis is limited to fatal cases among persons aged <65 years reported per state regulation, data on persons aged ≥65 years, who typically are at highest risk for severe influenza infections and death, are not included. Second, this midseason analysis might have resulted in underestimation of cumulative cases as well as morbidity and mortality rates when calculated across the season. Third, because reporting of ICU cases is voluntary, ascertainment of such cases might not be complete. Finally, the representativeness of the data might be limited by delayed reporting from some LHJs. The interpretations in this report might change as additional data become available.

Because weeks or months still remain in the 2013–14 influenza season, vaccination is still recommended, and persons who are in a group at higher risk for influenza complications, including adults aged <65 years with underlying medical conditions, are recommended to receive influenza vaccination as soon as possible. In the event of illness, persons at higher risk for influenza complications, whether vaccinated or not, should seek medical care promptly for assessment and potential early antiviral treatment.

What is already known on this topic?

The influenza A (H1N1)pdm09 virus has been the predominant circulating virus in the United States throughout the ongoing 2013–14 influenza season, resulting in high proportions of intensive-care unit (ICU) admissions and deaths among adults aged <65 years.

What is added by this report?

The 2013–14 influenza season in California has resulted in more ICU admissions and deaths associated with influenza virus infection than in any season since the 2009 H1N1 pandemic. Of fatal and ICU cases with laboratory-confirmed influenza occurring in persons aged <65 years, those aged 41–64 years with underlying medical conditions predisposing them to influenza complications have been disproportionately affected. Influenza vaccination and antiviral treatment have been underutilized in observed cases with overreliance on rapid diagnostic tests with poor sensitivity.

What are the implications for public health practice?

Early recognition of influenza illness and initiation of empiric antiviral treatment as soon as possible is recommended for persons with preexisting conditions that place them at high risk for influenza complications. Negative rapid influenza diagnostic test results should not be used to make clinical decisions on patients with influenza-like illness. Vaccination remains a critical public health tool in preventing severe influenza resulting in ICU admission or death.

## Figures and Tables

**TABLE:** Characteristics of influenza cases resulting in intensive-care unit (ICU) admission or death — California, September 29, 2013–January 18, 2014

	Deaths		ICU admissions	
		
Characteristic	No.	(%*)	No.	(%*)
**Overall**	**94**	**(100)**	**311**	**(100)**
**Sex**				
Male	46	(49)	172	(55)
Female	48	(51)	139	(45)
**Age group (yrs)**				
0–4	1	(1)	24	(8)
5–17	2	(2)	12	(4)
18–40	19	(20)	70	(23)
41–64	72	(77)	195	(63)
Unknown	0	—	10	(3)
**Race**				
American Indian	0	—	1	(0.3)
Asian	4	(4)	12	(4)
Black	6	(6)	16	(5)
Pacific Islander	0	—	2	(0.6)
White	64	(68)	162	(52)
Other	0	—	2	(0.6)
Unknown	20	(21)	116	(37)
**Ethnicity**				
Hispanic	30	(32)	57	(18)
Non-Hispanic	44	(47)	138	(44)
Unknown	20	(21)	116	(37)
**Preexisting medical condition**			N/A	
ACIP-influenza: Yes	74	(79)		
ACIP-influenza: No	6	(6)		
ACIP-influenza: Unknown	14	(15)		
**Obesity level**			N/A	
BMI ≥40 (morbidly obese)	11	(12)		
BMI 30–39 (obese)	16	(17)		
BMI <30	29	(31)		
Unknown BMI	38	(40)		
**Influenza type**				
Influenza A	94	(100)	303	(97)
Influenza B	0	—	8	(3)
**Influenza subtype**				
A (H1N1)pdm09	77	(82)	165	(53)
Pending or unknown	17	(18)	146	(47)
**Patient received antiviral therapy (n = 80)**			N/A	
Yes	62	(78)		
No	18	(23)		
**Antiviral start date on or before date of admission (n = 58)**			N/A	
Yes	27	(46)		
No	31	(54)		
**Antiviral therapy started ≤48 hours after symptom onset (n = 47)**			N/A	
Yes	8	(17)		
No	39	(83)		
**2013–14 influenza vaccination status** †			N/A	
Yes	6	(6)		
No	22	(23)		
Unknown or not reported in medical record	66	(70)		
**Rapid influenza antigen diagnostic test**			N/A	
Positive	14	(15)		
Negative	10	(11)		
Not performed or not reported in medical record	70	(74)		

**Abbreviations:** ACIP = Advisory Committee on Immunization Practices; BMI = body mass index; weight (kg) / (height [m])^2^; N/A = not available.

* Percentages might not sum to 100% because of rounding.

† ≥2 weeks before symptom onset.

## Footnotes

* The detailed timeline for changes to severe influenza reporting requirements beginning with the 2009 pandemic is as follows: Pediatric influenza deaths were reportable across the entire period. At the initial wave of the 2009 pandemic, case report forms were required for all probable and confirmed cases of pH1N1 virus infection, regardless of severity. On August 12, 2009, cases for hospitalized patients could be reported to the state as weekly aggregate numbers; individual case report forms were still required for ICU and fatal cases. On August 10, 2010, the 2009 influenza A (H1N1) pandemic was declared over, and the strain was no longer reportable as a novel influenza strain; ICU and fatal cases were voluntarily reportable and reporting of pediatric deaths resumed as prior to the pandemic. In August 2011, all influenza-related deaths among persons aged <65 years were made reportable to CDPH and reporting of ICU cases among persons aged <65 years remained voluntary.

† Three large California LHJs do not typically report ICU cases to CDPH in real time, and one LHJ does not report ICU cases; thus, only fatal cases from those populations are fully represented in this analysis.

§ Additional information available at http://www.cdc.gov/flu/professionals/diagnosis/rapidlab.htm.

¶ Additional information available at http://www.cdc.gov/flu/professionals/antivirals/antiviral-agents-flu.htm.

** Additional information available at http://www.cdc.gov/flu/professionals/antivirals/intravenous-antivirals.htm.

## References

[1] CDC (2013). Prevention and control of seasonal influenza with vaccines. Recommendations of the Advisory Committee on Immunization Practices—United States, 2013–2014. MMWR.

[2] Louie JK, Acosta M, Winter K (2009). Factors associated with death or hospitalization due to pandemic 2009 influenza A (H1N1) infection in California. JAMA.

[3] CDC (2014). FluView.

[4] CDC (2010). Prevention and control of influenza with vaccines: recommendations of the Advisory Committee on Immunization Practices (ACIP), 2010. MMWR.

[5] Chartrand C, Leeflang MMG, Minion J, Brewer T, Pal M (2012). Accuracy of rapid influenza diagnostic tests: a meta-analysis. Ann Intern Med.

[6] Uyeki TM, Prasad R, Vukotich C (2009). Low sensitivity of rapid diagnostic test for influenza. Clin Infect Dis.

[7] Writing Committee of the WHO Consultation on Clinical Aspects of Pandemic (H1N1) 2009 Influenza (2010). Clinical aspects of pandemic 2009 influenza A (H1N1) virus infection. New Engl J Med.

[8] Louie JK, Yang S, Acosta M (2012). Treatment with neuraminidase inhibitors for critically ill patients with influenza A (H1N1) pdm09. Clin Infect Dis.

[9] CDC (2011). Antiviral agents for the treatment and chemoprophylaxis of influenza. MMWR.

[10] Viasus D, Paño-Pardo JR, Pachón J (2011). Timing of oseltamivir administration and outcomes in hospitalized adults with pandemic 2009 influenza A (H1N1) virus infection. Chest.

